# Nitric Oxide Mediates Inflammation in Type II Diabetes Mellitus through the PPAR*γ*/eNOS Signaling Pathway

**DOI:** 10.1155/2020/8889612

**Published:** 2020-11-26

**Authors:** Hua Guo, Qinglan Zhang, Haipo Yuan, Lin Zhou, Fang-fang Li, Sheng-Ming Wang, Gang Shi, Maojuan Wang

**Affiliations:** ^1^Department of Clinical Laboratory, Hospital of Chengdu University of Traditional Chinese Medicine, Chengdu, China; ^2^Department of Endocrinology, Chongqing Hospital of Traditional Chinese Medicine, Chongqing, China; ^3^Department of Endocrinology, Hospital of Chengdu University of Traditional Chinese Medicine, Chengdu, China; ^4^Department of Ophthalmology, Huai'an Second People's Hospital, The Affiliated Huai'an Hospital of Xuzhou Medical University, Huai'an, Jiangsu Province, China; ^5^Department of Stomatology, Huai'an Second People's Hospital, The Affiliated Huai'an Hospital of Xuzhou Medical University, Huai'an, Jiangsu Province, China; ^6^Department of Pharmacy Services, Hospital of Chengdu University of Traditional Chinese Medicine, Chengdu, China; ^7^Department of Outpatient, Hospital of Chengdu University of Traditional Chinese Medicine, Chengdu, China

## Abstract

Inflammation accounts for the process of type II diabetes mellitus (T2DM), the specific mechanism of which is still to be elucidated yet. Nitric oxide (NO), a critical inflammation regulator, the role of which is the inflammation of T2DM, is rarely reported. Therefore, our study is aimed at exploring the effect of NO on the inflammation in T2DM and the corresponding mechanism. We analyzed the NO levels in plasma samples from T2DM patients and paired healthy adults by Nitric Oxide Analyzer then measured the expression of inflammatory cytokines (C-reactive protein, heptoglobin, IL-1*β*, TNF-*α*, IL-6) in insulin-induced HepG2 cells treated with NO donor or NO scavenger, and the PPAR*γ*, eNOS, C-reactive protein, heptoglobin, IL-1*β*, TNF-*α*, and IL-6 levels were detected by RT-PCR and western blot in insulin-induced HepG2 cells transfected with si-PPAR*γ*. The results showed that excess NO increased the inflammation marker levels in T2DM, which is activated by the PPAR*γ*/eNOS pathway. These findings will strengthen the understanding of NO in T2DM and provide a new target for the treatment of T2DM.

## 1. Introduction

Type II diabetes mellitus (T2DM) is a common chronic metabolic and endocrine disease characterized by insulin resistance and *β*-cell dysfunction. The high prevalence of T2DM is a serious public health event over the world [1]. According to the latest data given by the Chinese Diabetes Society (CDS), the incidence of T2DM in adults over the age of 18 in China is increasing annually and up to 10.4% by 2019 [2]. Studies have shown that the onset of T2DM is associated with a complex interaction between environmental and genetic factors [3, 4], and the specific pathogenesis of the disease has yet to be elucidated. It has been reported that chronic inflammatory responses may be involved in the process of insulin resistance and *β*-cell dysfunction, which adds the risk of developing T2DM [5, 6]. C-reactive protein can lead to insulin resistance and is an important indicator of the level of inflammation in T2DM [5, 7]. Haptoglobin is an important indicator of liver inflammation, and it is shown that the haptoglobin 2-2 genotype might increase the risk of cardiovascular disease in diabetic patients [8, 9]. Inflammatory factors such as TNF-*α*, IL-1*β*, and IL-6 have been clarified to cause insulin resistance by inhibiting insulin signaling [10, 11]. Therefore, actively exploring the mechanisms of inflammation in T2DM can provide new ideas for the treatment of the disease.

PPAR*γ* belongs to a family of peroxisome proliferator-activated nuclear receptors that regulate the expression of multiple genes involved in the regulation of lipid/glucose/amino acid metabolism, cell proliferation/differentiation, and inflammation, suggesting that it may play important roles in many diseases [12]. Thiazolidinedione, a target drug of PPAR*γ*, has potent insulin-sensitizing effects and is used to treat T2DM, but the drug will produce side effects such as weight gain, liver damage, and cardiovascular risk and is gradually declining in clinical use [13, 14]. Thus, it is extremely important to clarify the mechanism of PPAR*γ* in T2DM. In recent years, studies have told that PPAR*γ* regulates the expression of inflammatory pathways. For example, GMG-43AC antagonist can be applied to treat acne because it activates PPAR*γ* to inhibit inflammation [12]. PPAR*γ*-mediated upregulation of CD36 is involved in the regulation of microglial activation and phenotype and promotes phagocyte-cell proliferation of apoptotic cells, thereby promoting the recovery of postischemic inflammation [15]. Moreover, PPAR*γ* activation is essential in the suppression of intestinal inflammatory response [16]. In T2DM, the detailed mechanism by which PPAR*γ* regulates inflammation remains to be refined.

Nitric oxide (NO), a free radical molecule with pathophysiological functions, is extensively studied in inflammation. NO has anti-inflammatory and proinflammatory effects, which are correlated with its concentration [17]. Excess NO will damage cells and organs and interacts with intermediate components of reactive oxygen species in cells to induce inflammation [18]. Studies have indicated that the NO/inflammatory signaling pathway is associated with the development of depression [19], colon cancer [20], lung squamous cell carcinoma [18], and diabetes [21]. Endothelial nitric oxide synthase (eNOS) is a kind of nitric oxide synthase that induces NO production, occupying the dominant role in regulating NO activity [22]. Previous studies have shown that the PPAR*γ*/eNOS pathway regulates hypertension [23], ischemia/reperfusion-induced acute kidney injury [24], steatohepatitis [25], ischemia/reperfusion-induced liver injury [26], and so on. However, it is not clear whether the PPAR*γ*/eNOS pathway mediates the inflammatory process in T2DM.

Therefore, we put forward the hypothesis that NO may regulate inflammation in T2DM through the PPAR*γ*/eNOS pathway and validated in the present study. Our findings may provide a new treatment target for T2DM.

## 2. Materials and Methods

### 2.1. Blood Collection from T2DM Patients

The study was approved by the ethics committee of Huai'an Second People's Hospital and Hospital of Chengdu University of Traditional Chinese Medicine. Fifty-five T2DM patients and fifty healthy adults in this study signed informed consent forms and began to sample blood at 8:30 am in the morning under a fasting state (no food and water absorption for at least 8 h before sampling). The whole blood samples were collected and placed in a heparin anticoagulation tube (BD, USA), then centrifuged at 3500 g at 4°C for 10 min. The achieved uppermost layer is the plasma layer, which should be stored at -80°C for subsequent testing.

### 2.2. Cell Culture

HepG2 cells were purchased from the Cell Bank of the Chinese Academy of Sciences (Shanghai, China); they were cultured in 96-well plates in RPMI1640 medium (Gibco, NY, USA) with 10% fetal bovine serum (Gibco, USA) and 1% penicillin-streptomycin (Gibco, NY, USA) and allowed to grow to logarithmic growth phase for subsequent studies after a successful recovery. To construct a cell model of T2DM, insulin (Gibco, NY, USA) was first diluted in RPMI1640 complete medium to a final concentration of 10^−6^ mol/L. 200 *μ*L of insulin preparation solution was added to each well in the model group, and an equal amount of RPMI1640 complete medium was added to each well in the control group; all cells were cultured for 48 h [27]. In the process of cultivation, cells were incubated in an incubator (37°C, 5% CO_2_).

### 2.3. RNA Interference

HepG2 cells were transfected with 100 pmol PPAR*γ*siRNA (sense, 5′-UAAAUGUCAGUACUGUCGGUUU-3′, antisense, 5′-CCGACAGUACUGACAUUUAUU-3′) by using the Amaxa Lonza Cell Line Nucleofector® Kit (Lonza, Germany) according to the manufacturer's instructions, and an equal amount of nonspecific siRNA (sense, 5′-UUCUCCGAACGUGUCACGU-3′; antisense, 5′-(ACGUGACACGUUCGGAGAA-3′) was transfected with HepG2 cells as a negative control. After incubation for 24 h, they were used for western blot analysis.

### 2.4. The Measurement of Nitric Oxide

Due to the fact that NO has a short half-life and is not easy to directly detect, the concentration of its stable metabolites nitrite and nitrate can represent the levels of NO [28]. In the present study, nitrite and nitrate in plasma and cells were measured using the Nitric Oxide Analyzer 280i (GE, USA), and there is no need for pretreatment samples before testing. Each sample was tested 3 times.

### 2.5. ELISA Assay

The levels of IL-1*β*, TNF-*α*, and IL-6 in HepG2 cells were detected using an ELISA kit (SPI-BIO, Bertin Pharma, France), and each experiment was performed 3 times according to the manufacturer's instructions.

### 2.6. Real-Time Quantitative PCR

Total RNA was isolated from cells using TRIzol reagent (Life Technologies, NY, USA) then reverse transcribed into single-stranded cDNA using a Prime ScriptTMRT kit (Takara, Dalian, China). Real-time PCR equipment (7500 Real-Time PCR System, USA) was to detect the expression of C-reactive protein, haptoglobin, eNOS, and PPAR*γ*. The expression levels were analyzed by the -*ΔΔ*2Ct method, and GADPH (5′-AGGTCGGAGTCAACGGATTT-3′ (forward) and 5′-TAGTTGAGGTCAATGAAGGG-3′ (reverse)) expression levels were used as the reference standard.

### 2.7. Western Blot

After HepG2 cells were treated with RIPA lysis buffer (Sigma, USA), 30 *μ*g of total protein was isolated on 12% SDS-PAGE and transferred to PVDF membranes. Membrane blotting was first blocked with 5% bovine serum albumin (Sigma, USA) for 1 h then incubated with primary antibody (anti-C-reactive protein, antihaptoglobin, antieNOS, anti-PPAR*γ*) overnight at 4°C, followed by incubation with horseradish peroxidase for 2 h at room temperature. The relative protein expression levels were normalized to GAPDH.

### 2.8. Data Analysis

All statistics were analyzed using GraphPad Prism 7.0 software (USA), and all experimental data were expressed as mean ± SD. Whether the expression of nitrite, nitrate, and NO differed between the different groups was obtained by *t*-test analysis. And *p* < 0.05 indicates statistical significance.

## 3. Results

### 3.1. High NO Production in Plasma in T2DM Patients

To evaluate the effect of NO in the T2DM, we firstly examined the levels of nitrite and nitrate in plasma between 55 T2DM patients and paired 50 healthy adults (control group). The clinical data of volunteers in the study is shown in Table 1.

**Figure 1:**
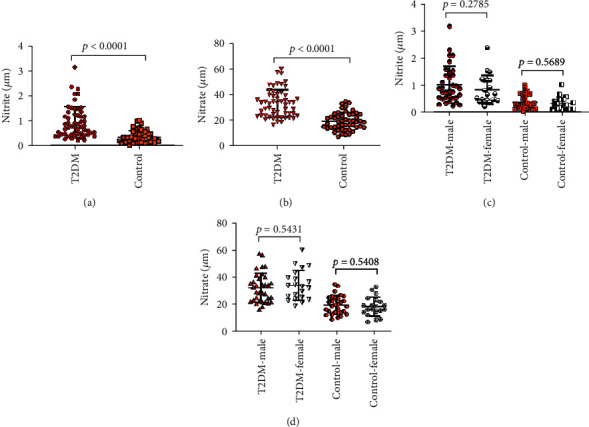
The nitrite and nitrate levels in plasma in the T2DM group and control group.

The concentration of plasma nitrite in the T2DM group was significantly higher than the concentration of plasma nitrate in the control group (*p* < 0.0001), and the plasma nitrate levels between the T2DM group and control group were evidently different (*p* < 0.0001), which was found in Figures 1(a) and 1(b). Else, as shown in Figures 1(c) and 1(d), we also found that there were no significant gender differences in plasma nitrite or nitrate contents in the T2DM group and control group. This status revealed the abnormal NO expression in T2DM patients.

### 3.2. NO Promoted Inflammation in Insulin-Induced HepG2 Cells

**Figure 2:**
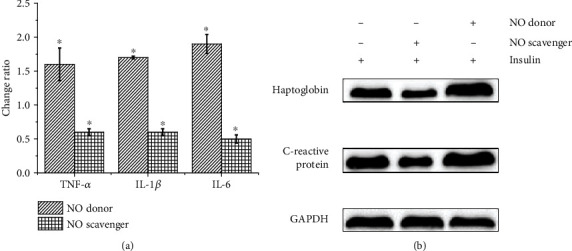
The inflammatory factor levels in HepG2 cells. (a) The ratio of TNF-*α*, IL-1*β*, and IL-6 contents in insulin-induced HepG2 cells treated with NO donor or NO scavenger and TNF-*α*, IL-1*β*, and IL-6 contents in untreated insulin-induced HepG2 cells. (b) Western blot detects the C-reactive protein and heptoglobin expression in insulin-induced HepG2 cells after different treatments.

NO regulates inflammation, and inflammation promotes the development of T2DM. To investigate whether NO is involved in inflammation in T2DM, we first stimulated HepG2 cells with insulin in vitro to obtain a cell model of T2DM in this study. The cells were then treated with the NO donor DEA (Sigma, USA) and NO scavenger 2-(4-carboxyphenyl)-4, 4, 5, 5-tetramethylimidazoline-1-oxyl-3-oxide (Sigma, USA), respectively. The concentration of IL-1*β*, TNF-*α*, and IL-6 is expressed in Table 2. Compared with insulin-induced HepG2 cells, the levels of the inflammatory factors IL-1*β*, TNF-*α*, IL-6, C-reactive protein, and heptoglobin in the cells supplied with NO donor were significantly increased, while the levels of these inflammatory factors were significantly decreased in cells treated with NO scavenger, as shown in Figure 2. The above results indicate that the level of NO correlates with the degree of T2DM inflammation.

### 3.3. PPAR*γ*/eNOS/NO Signaling Is Associated with Inflammation in T2DM

**Figure 3:**
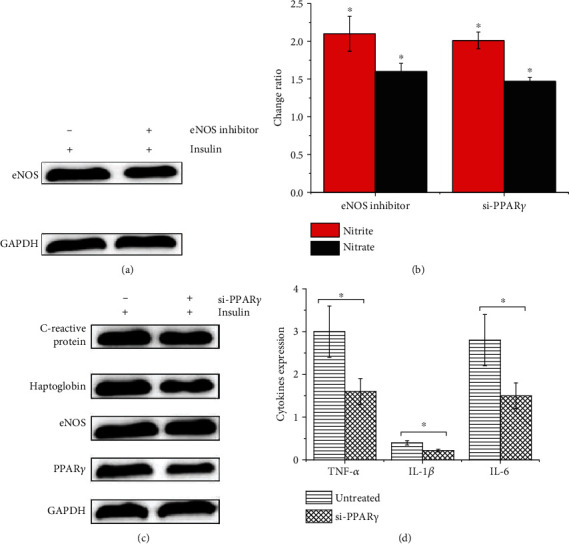
The eNOS, NO, PPAR*γ*, inflammatory factors levels in HepG2 cells. (a) The expression of eNOS in insulin-induced HepG2 cells. (b) The change of nitrite and nitrate in insulin-induced HepG2 cells treated with eNOS inhibitor or si-PPAR*γ*. (c) The effect of PPAR*γ* inhibition on the expression of eNOS, PPAR*γ*, C-reactive protein, and heptoglobin expression in insulin-induced HepG2 cells. (d) The IL-1*β*, TNF-*α*, and IL-6 levels in insulin-induced HepG2 cells and insulin-induced HepG2 cells transfected with si-PPAR*γ*.

To investigate the mechanism of the development of inflammation in T2DM, we detected the expression of eNOS and NO in vitro. As shown in Figures 3(a) and 3(b), the expression of eNOS and NO was significantly reduced in insulin-treated HepG2 cells after the addition of the NOS inhibitor L-NAME (Cayman, USA), which suggested that the abnormal expression of NO in T2DM might be related to the abnormal expression of eNOS. Considering that PPAR*γ* can modulate the level of diabetic inflammation, the PPAR*γ*/eNOS pathway plays an important role in several diseases. In the present study, insulin-induced HepG2 cells transfected with PPAR*γ* siRNA were found to significantly decrease the expression of PPAR*γ*, eNOS, and NO in Figures 3(b) and 3(c). In addition, Figures 3(c) and 3(d) showed that the inhibition of the PPAR*γ* expression in HepG2 cells prompted a significant decrease in the expression levels of IL-1*β*, TNF-*α*, IL-6, C-reactive protein, and heptoglobin. These results suggest that inflammation in T2DM may be associated with the PPAR*γ*/eNOS/NO pathway.

## 4. Discussion

The inflammatory response can drive the pathological process of T2DM by leading to deleterious effects on tissue function and insulin resistance [10, 29], so exploring the mechanisms of inflammation in T2DM may be extremely important for the treatment of T2DM that currently lacks an effective cure. NO plays significant roles in the inflammatory process and is a potential target for the treatment of inflammatory diseases [30]. In this study, plasma samples collected from 55 T2DM patients were analyzed by Nitric Oxide Analyzer for the first time, and the sample numbers were higher than the previous publications; the results showed higher levels of nitrite and nitrate in the plasma of the T2DM patients. Similarly, it also showed that abnormal levels of nitrogen oxides in plasma, serum and urine samples of T2DM patients before [31, 32]. It suggests that NO may play an important effect in the pathogenesis of T2DM.

In vitro experiment revealed that the concentration of inflammatory factors (C-reactive protein, heptoglobin, IL-1*β*, TNF-*α*, IL-6) was significantly increased in insulin-induced HepG2 cells after NO donor treatment, while the levels of inflammatory factors were decreased in insulin-induced HepG2 cells stimulated with NO scavenger. The results support that NO may take part in the inflammatory process in T2DM patients. To investigate the mechanism of NO on inflammation in T2DM, we investigated and conducted further studies. Previous studies have shown that PPAR*γ* hyperglycosylation modification induces endothelial insulin resistance and dysfunction associated with diabetic vascular complications by regulating the eNOS-NO pathway [33]. PPAR*γ* provides assistance to the expression of eNOS [24], which induces NO production. However, whether NO can regulate T2DM inflammation through the PPAR*γ*-eNOS signaling pathway is currently unclear.

To test the hypothesis, this study detected the expression of eNOS and PPAR*γ* in vitro and found that the expression of eNOS and NO decreased after the treatment of insulin-induced HepG2 cells with NOS inhibitor. Inhibiting the expression of PPAR*γ* in insulin-induced HepG2 cells significantly decreased the levels of PPAR*γ*, eNOS, and NO, and the levels of C-reactive protein, heptoglobin, IL-1*β*, TNF-*α*, and IL-6 were significantly reduced. Therefore, the expression of T2DM inflammation may be regulated through the PPAR*γ*/eNOS pathway-mediated expression of NO.

In summary, this study explored the link between NO and inflammation through insulin-induced HepG2 cells, which provides a potential therapeutic target for the possible treatment of T2DM.

## Figures and Tables

**Table 1:** Clinical data in the T2DM group and control group.

	T2DM (*n* = 55)			Control (*n* = 50)		*p*
Age (yrs)	34-50			32-51		
Gender (M/F)	34/21			30/20		
Plasma nitrite (*μ*M)	0.9728 ± 0.6274			0.3779 ± 0.2579		<0.0001
Plasma nitrate (*μ*M)	33.1243 ± 10.7757			19.3209 ± 6.8687		<0.0001

	M	F	*p*	M	F	*p*
Plasma nitrite (*μ*M)	1.0454 ± 0.6776	0.8551 ± 0.5305	0.2785	0.3951 ± 0.2680	0.3521 ± 0.2465	0.5689
Plasma nitrate (*μ*M)	32.4212 ± 10.7574	34.2627 ± 10.9713	0.5431	19.8127 ± 6.8145	18.5833 ± 7.0596	0.5408

**Table 2:** The TNF-*α*, IL-1*β*, and IL-6 contents in insulin-stimulated HepG2 cells.

Cytokines (ng/mL)	Insulin-stimulated HepG2 cells	Insulin-stimulated HepG2 cells+NO donor	Insulin-stimulated HepG2 cells+NO scavenger
TNF-*α*	0.30 ± 0.06	0.48 ± 0.09	0.18 ± 0.04
IL-1*β*	0.05 ± 0.01	0.09 ± 0.02	0.03 ± 0.01
IL-6	0.28 ± 0.06	0.53 ± 0.70	0.14 ± 0.05

## Data Availability

All data are available upon request.
